# Two infant cases of intraperitoneal arterial hemorrhage due to a duplication cyst: a case report

**DOI:** 10.1186/s40792-020-00820-1

**Published:** 2020-03-21

**Authors:** Hiroaki Fukazawa, Keisuke Kajihara, Yasuhiro Kuroda, Yuki Fujieda, Kotaro Uemura, Yuki Takeuchi, Yoshitomo Samejima, Insu Kawahara, Keiichi Morita, Tamaki Iwade, Kosaku Maeda

**Affiliations:** 1 Division of Pediatric Surgery, Department of Surgery, Kobe University Graduate School of Medicine, Kobe, Japan; 2 Department of Pediatric Surgery, Kobe Children’s Hospital, 1-6-7 Minatojima-minami, Chuo-ku, Kobe, 650-0047 Japan; 3 Department of Pediatric Surgery, Kyusyu University, Fukuoka, Japan

**Keywords:** Arterial hemorrhage, Duodenum, Infant, Intestinal duplication, Intraperitoneal hemorrhage, Pseudopancreatic cyst

## Abstract

**Background:**

Intraperitoneal arterial hemorrhage without trauma is extremely rare. We report two infant cases of intraperitoneal arterial hemorrhage due to intestinal duplication.

**Case presentation:**

In case 1, a 2-month-old girl experienced sudden intraperitoneal hemorrhage from the middle colic artery with no apparent trauma. Hemostasis was achieved with suturing of the hemorrhage point, but the cause of hemorrhage was still unknown. Computed tomography after the first operation revealed a duodenal duplication cyst and a pseudopancreatic cyst. Percutaneous drainage of the pseudopancreatic cyst was performed, and the contents had high pancreatic amylase. As the size of the duodenal duplication cyst also decreased with this drainage, we suspected that the duodenal duplication cyst was connected to the pseudopancreatic cyst and the arterial hemorrhage. We hypothesized that the pancreatic juice inside the duplication cyst leaked into the intraperitoneal cavity and caused rupture of the arterial wall. Therefore, marsupialization of the duodenal duplication was performed to evacuate the pancreatic juice contained in the cyst toward the native duodenum. The postoperative course was uneventful.

In case 2, a 6-month-old boy experienced sudden intraperitoneal hemorrhage without trauma. The hemorrhage site was identified as the ileocecal artery, and hemostasis was achieved with sutures. Tissue near the hemorrhage point was biopsied, because the cause of arterial wall rupture was still unknown. The biopsied tissue was found to be intestinal mucosa. The patient had recurrent abdominal pain after the first operation, and computed tomography showed a duplication cyst located near the hemorrhage point. Therefore, we resected the intestinal duplication. Pathology results showed that the intestinal duplication contained intestinal mucosa, ectopic gastric mucosa, and pancreatic tissue. The postoperative course was uneventful.

**Conclusion:**

Intraperitoneal arterial hemorrhage without trauma is an extremely rare condition, and identifying its cause is difficult. To our knowledge, this is the first report of intraperitoneal arterial hemorrhage due to intestinal duplication. In cases of unexplained intraperitoneal arterial hemorrhage in infants, intestinal duplication near the hemorrhage point should be suspected.

## Background

Sudden intraperitoneal hemorrhage in infants is a rare condition, except in trauma cases. In fact, no published reports about spontaneous intraperitoneal arterial hemorrhage in infants could be found. We experienced two infant cases of sudden intraperitoneal arterial hemorrhage without trauma. In both cases, the cause of arterial wall rupture was associated with intestinal duplication.

Alimentary duplications are rare congenital anomalies that can occur in any portion of the gastrointestinal tract from the mouth to the anus [1]. Intestinal duplications have a well-developed layer of smooth muscle and mucosal membrane lining derived from some parts of the intestinal tract. In some cases, ectopic gastric mucosa or pancreatic tissue is present. Most, but not all, lesions are attached to the native intestinal tract [2]. It is well known that intestinal hemorrhage is caused by intestinal duplication. However, in our two cases, the intraperitoneal arterial hemorrhage occurred due to intestinal duplication. Herein, we described in detail the cases of intraperitoneal arterial hemorrhage caused by intestinal duplication in two infants.

## Case presentation

### Case 1

**Fig. 1:**
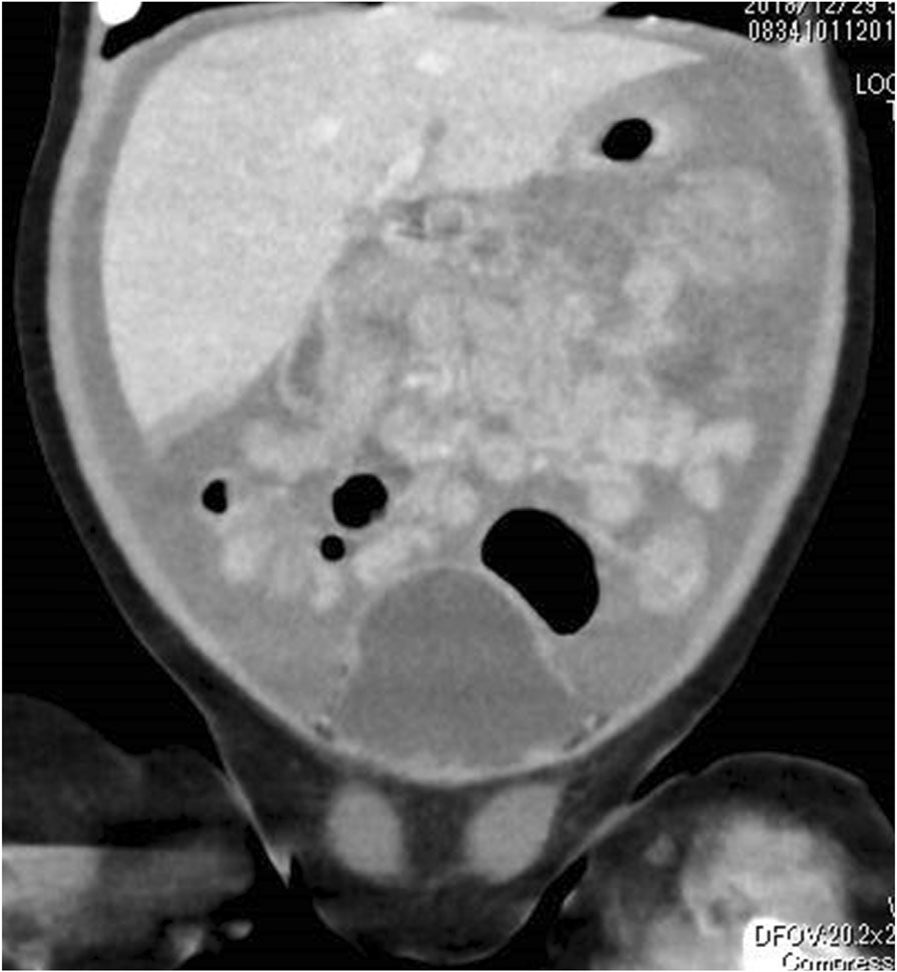
Computed tomography (CT), case 1. CT reveals intraperitoneal hemorrhage, but without extravasation

**Fig. 2:**
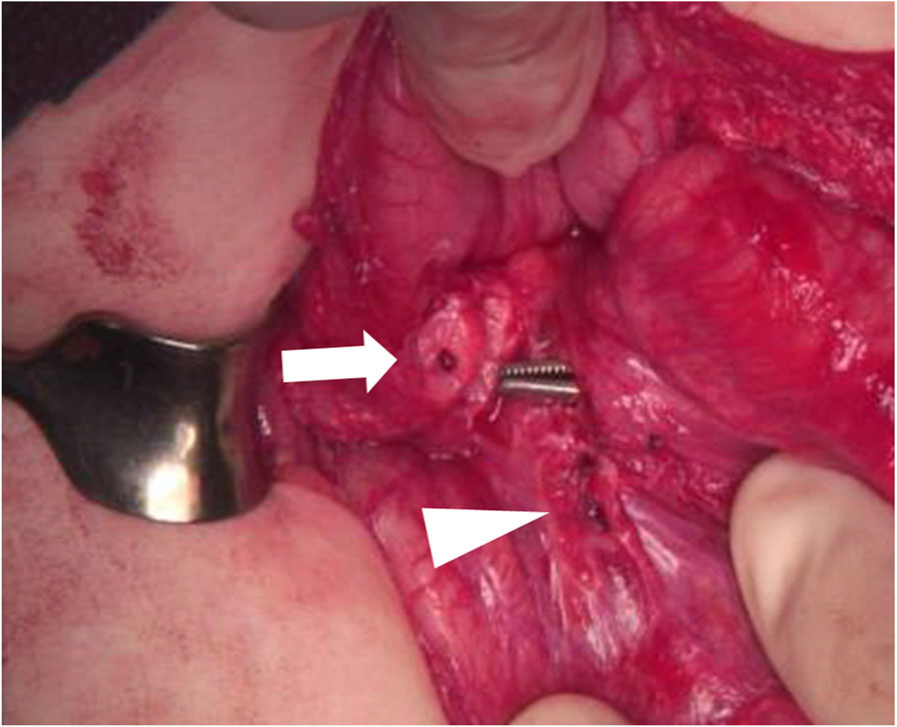
Intra-operative view, case 1. The white triangle indicates the hemorrhage point. The white arrow indicates ectopic pancreatic tissue with a small hole

**Fig. 3:**
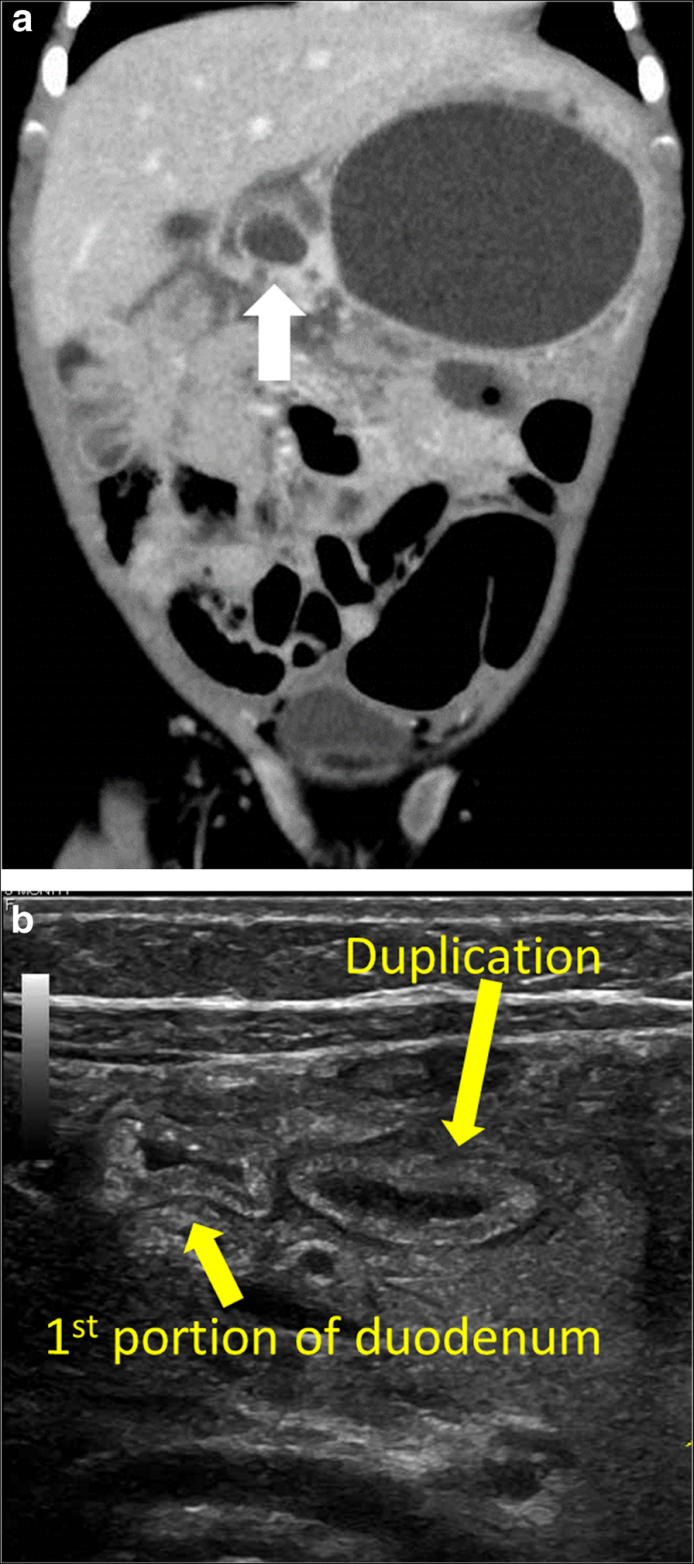
Post-surgical imaging, case 1. **a** Computed tomography (CT) reveals a large pseudopancreatic cyst and a duodenal duplication cyst (white arrow) attached to the first portion of the native duodenum. **b** Ultrasonography (sagittal view) shows duplication at the first portion of the duodenum with hyperechoic mucosa surrounded by hypoechoic muscular layers

A 2-month-old female infant presented to another hospital with acute circulatory failure and severe abdominal distention. Severe anemia and intraperitoneal echo-free space was detected, and she was transferred to our hospital for emergent operation. There were no indications of trauma on her body surface. Computed tomography (CT) revealed intraperitoneal hemorrhage, but extravasation and any other findings were not detected (Fig. 1). Blood transfusion was performed; however, circulation was not stable, and emergent laparotomy was performed. A large amount of bloody ascites was present, but the active hemorrhage point was not detected. A fistula with clot was identified on the transverse mesocolon near the Treitz ligament. Therefore, it was suspected that the hemorrhage occurred around the pancreas. When the bursa omentalis was dissected and the transverse mesocolon was explored, sudden arterial hemorrhage reoccurred from the middle colic artery. Hemostasis was achieved with sutures. Ectopic pancreatic tissue with a small hole was detected at the first portion of the duodenum (Fig. 2). The ectopic tissue was not resected, because it was not suspected to be associated with the arterial wall rupture. The patient almost fully recovered; however, intraperitoneal re-hemorrhage with circulatory failure occurred 1 month later. Hemostasis occurred spontaneously. After her condition stabilized, CT revealed a large pseudopancreatic cyst and another small cyst attached to the first portion of the duodenum (Fig. 3a). Ultrasonography showed the small cystic lesion with hyperechoic mucosa surrounded by hypoechoic muscular layers (Fig. 3b), and a duodenal duplication cyst was diagnosed. We considered that the duodenal duplication cyst was actually the ectopic pancreatic tissue and small hole identified at the first operation.

Percutaneous drainage of the pseudopancreatic cyst was performed, and high pancreatic amylase was detected in the contents. As the size of the pseudopancreatic cyst was decreased due to drainage, the size of duodenal duplication also decreased.

**Fig. 4:**
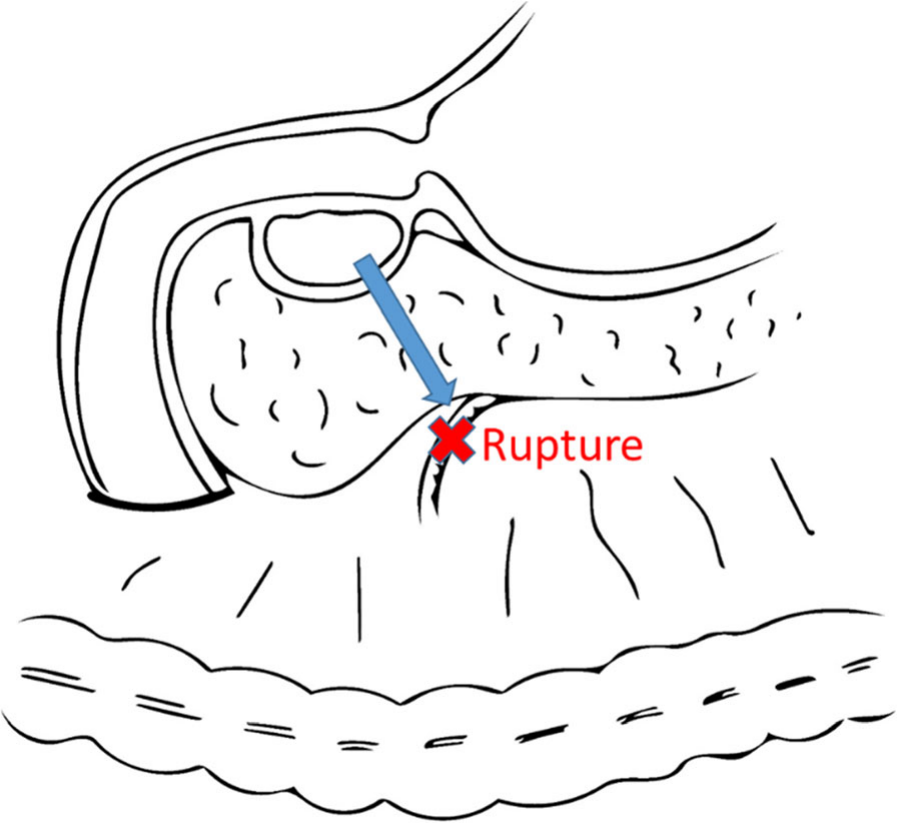
Schema of case 1. Pancreatic juice contained in the duodenal duplication leaked out and injured the arterial wall

We theorized that the duodenal duplication cyst with ectopic pancreatic tissue was originally present, and the pancreatic juice produced by the ectopic pancreatic tissue flowed into the duodenal duplication cyst. Thus, the duplication cyst gradually enlarged and ruptured, allowing the pancreatic juice inside the duplication cyst to leak into the intraperitoneal cavity and rupture the arterial wall (Fig. 4).

**Fig. 5:**
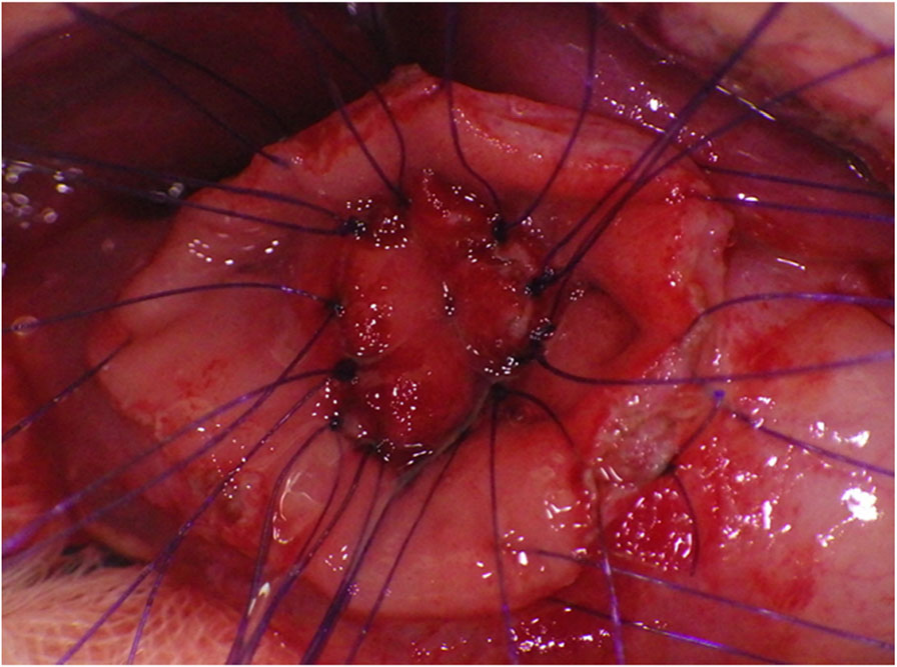
Intra-operative view, case 1. Marsupialization of the duodenal duplication is performed inside the native duodenum

Therefore, we performed marsupialization of the duplication inside the native duodenum lumen, and the contents of the duplication cyst were drained inside the duodenum (Fig. 5). After this operation, the patient was followed for 1 year with no complaints or symptoms.

### Case 2

**Fig. 6:**
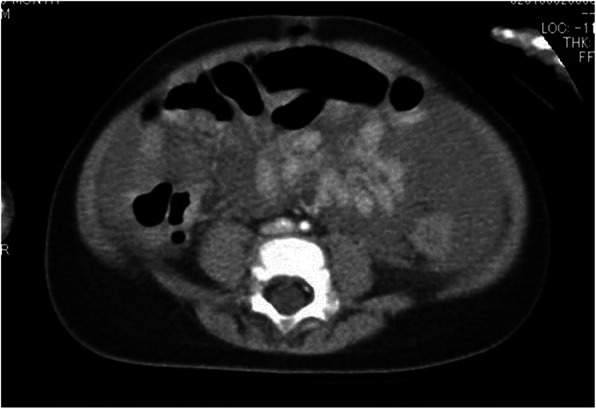
Initial computed tomography (CT), case 2. CT shows massive bloody ascites

**Fig. 7:**
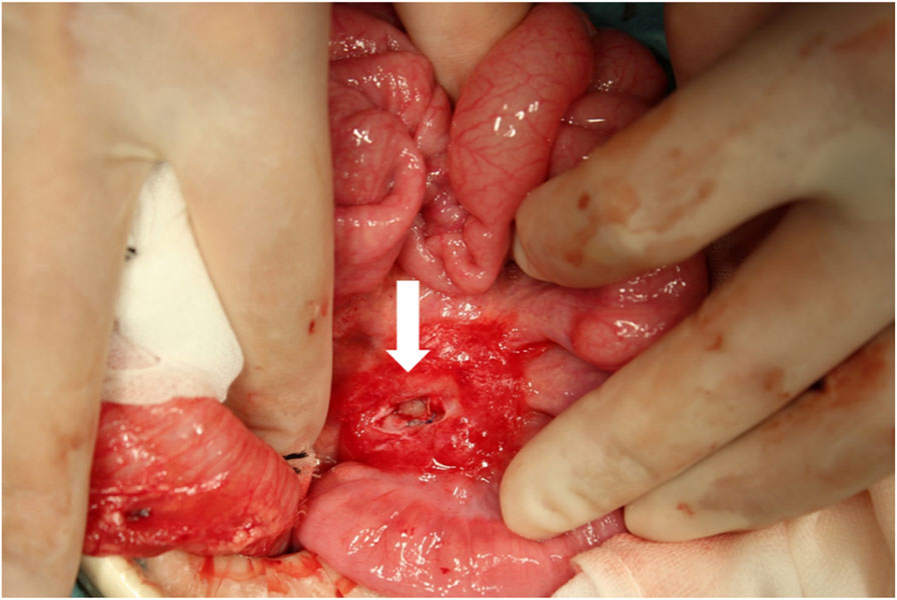
Intra-operative view, case 2. The hemorrhage point (white arrow) is located on the ileal mesentery. The tissue near the hemorrhage point was biopsied

**Fig. 8:**
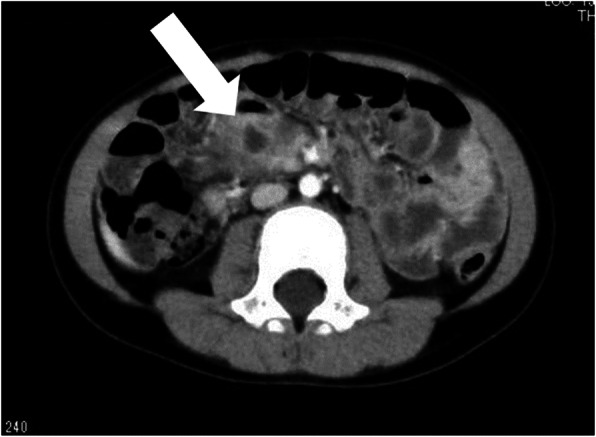
Follow-up computed tomography (CT), case 2. CT shows the remaining duplication cyst around the end of the ileum (white arrow)

A 6-month-old boy presented with acute circulatory failure with severe abdominal distention. Intraperitoneal echo-free space was detected. There were no indications of trauma on his body surface. CT revealed intraperitoneal hemorrhage, but extravasation and any other findings were not detected (Fig. 6). His circulation was unstable, and emergent laparotomy was performed. A large amount of bloody ascites was present, and a clot was detected on the mesentery around the ileocecal artery. When the clot was removed, hemorrhage from the ileocecal artery was immediate. Hemostasis was achieved with sutures (Fig. 7). We biopsied the tissue around the hemorrhage point, because we could not identify any cause of the arterial hemorrhage. Pathological results indicated that the tissue near the hemorrhage point was intestinal mucosa. We suspected that the rupture of the ileocecal artery was associated with the intestinal duplication located at the ileal mesentery. The patient was kept under close observation, and he had recurrent abdominal pain at 3 months postoperatively. CT showed a remaining duplication cyst around the end of the ileum (Fig. 8). We resected the intestinal duplication and small part of the ileum near the duplication cyst. Pathological results indicated that the intestinal duplication contained intestinal mucosa, ectopic gastric mucosa, and pancreatic tissue. After resection of the intestinal duplication, the patient was symptom-free at the 1-year follow-up.

## Discussion

Intraperitoneal hemorrhage without trauma is a rare condition in infants. To the best of our knowledge, intraperitoneal arterial hemorrhage in an infant has not been reported. We experienced two of such cases due to intestinal duplication.

In case 1, duodenal duplication was the cause of arterial hemorrhage. Duodenal duplication is rare. It represents only 2 to 12% of digestive tract duplications [3–8]. Chen et al. reviewed 47 cases of duodenal duplications [9]. Pancreatitis occurred in 53% of the cases. Moreover, their report showed that 29% of these duplication cysts were connected to the pancreaticobiliary ducts, and 2 cysts had aberrant ducts to the pancreatic head, supporting the theory that pancreatic juice can flow into the duodenal duplication in some cases. Marsupialization was performed in 40.4% (19/47) of their cases, and all had uneventful recoveries. This data suggests that marsupialization is an adequate treatment for duodenal duplication. In our first case, it is suspected that pancreatic juice leaked into the abdominal cavity and injured the middle colic artery wall. Marsupialization of the duodenal duplication allows pancreatic juice from any pancreatic duct to be evacuated into the native duodenum.

In our second case, the duplication cyst at the ileac mesentery contained ectopic gastric mucosa and pancreatic tissue. Ulceration due to the ectopic gastric mucosa may have penetrated to the ileocecal artery. On the other hand, the duplication cyst may have contained pancreatic juice from the ectopic pancreatic tissue, which then leaked into the ileocecal artery and caused hemorrhage. In any case, the duplication cyst is strongly associated with the arterial rupture.

It was very difficult to diagnose the cause of hemorrhage in our two cases, because we could not identify the intestinal duplications during the first CT performed preoperatively and there were no remarkable operative findings at the time of hemostasis. The duplication cyst might have shrunken at the time of intraperitoneal hemorrhage, because the pancreatic juice was evacuated. After hemostasis, the pancreatic juice flowed into the duplication cyst again, allowing its visualization through CT or ultrasonography.

Moreover, no symptoms were identified before arterial rupture in both cases. We hypothesized that the rate of pancreatic juice leakage from the duplication cyst was very low. Therefore, only the tissue around the duplication cyst may have been gradually damaged without symptoms, and arterial rupture suddenly occurred on one occasion.

## Conclusion

Intraperitoneal arterial hemorrhage without trauma is extremely rare, and it is difficult to identify its cause during hemostasis surgery. Intestinal duplication near the hemorrhage point should be considered as a potential cause of arterial wall rupture in intraperitoneal arterial hemorrhage.

## Data Availability

The data are not available for public access because of patient privacy concerns but are available from the corresponding author on reasonable request.

## Abbreviations

CT: Computed tomography
