# Correction to: Two infant cases of intraperitoneal arterial hemorrhage due to a duplication cyst: a case report

**DOI:** 10.1186/s40792-020-01034-1

**Published:** 2020-10-08

**Authors:** Hiroaki Fukuzawa, Keisuke Kajihara, Yasuhiro Kuroda, Yuki Fujieda, Kotaro Uemura, Yuki Takeuchi, Yoshitomo Samejima, Insu Kawahara, Keiichi Morita, Tamaki Iwade, Kosaku Maeda

**Affiliations:** 1 Division of Pediatric Surgery, Department of Surgery, Kobe University Graduate School of Medicine, Kobe, Japan; 2 Department of Pediatric Surgery, Kobe Children’s Hospital, 1-6-7 Minatojima-minami, Chuo-ku, Kobe, 650-0047 Japan; 3 Department of Pediatric Surgery, Kyusyu University, Fukuoka, Japan

## Correction to: Surg Case Rep 6:55 (2020)  https://doi.org/10.1186/s40792-020-00820-1

Following publication of the original article [1], the authors identified an error in the author name of Hiroaki Fukuzawa.

The incorrect author name is: Hiroaki Fukazawa.

The correct author name is: Hiroaki Fukuzawa.

The author group has been updated above.
